# Supplementary material for: Digital transformation, dynamic capabilities and new quality productive forces: Empirical data from listed Chinese manufacturing companies
Source: PLoS One. 2026 Mar 24;21(3):e0342917. doi: 10.1371/journal.pone.0342917 (PMC13012489; doi:10.1371/journal.pone.0342917)
Supplement: Attachment — Submitted filename: Response to Reviewers.docx [file pone.0342917.s002.docx]

**Responses to editor (original comments by editor are in blue color)**

We sincerely appreciate your valuable suggestions during the review process, which have been of great significance in enhancing the quality of our manuscript. We are particularly grateful for your meticulous attention to the transparency of data sources, the clarity of variable definitions, and the uniqueness of research contributions. These suggestions not only helped us identify the areas for improvement but also deepened our understanding of the rigor and standardization in academic research. We have revised each of your comments and refined the manuscript accordingly. The revised parts are highlighted in yellow in the manuscript. Below are our detailed responses to each of your comments.

**1.Comment:** Please clearly state the source of data for each variable utilized in your manuscript.

**1.Reply:** In response to the revision suggestions, in Section 3.1 Sample Selection and Data Sources, we have added detailed explanations of the data sources for the key variables such as digital transformation, new quality productive forces (Nqpf), and dynamic capabilities. We have not only specified the data sources for the specific indicators used to assess the level of Nqpf but also provided the data sources for the three dimensions of dynamic capabilities in enterprises (innovation capability, absorptive capability, and adaptive capability). At the same time, we have also given a detailed account of the data sources for the control variables, ensuring the reproducibility of the research.

**2.Comment:** Please provide a clear definition for each of the variables utilized in your manuscript, such that it is clear how they were derived.

**2.Reply:** Thank you for your valuable suggestions. We have clearly defined all the variables. In the first paragraph of the introduction, we defined the concept and characteristics of Nqpf. In section 2.1, " Digital transformation of manufacturing and development of Nqpf", we provided a detailed definition of the concept of digital transformation. In section 2.2, "The mediating role of dynamic capabilities", we also gave detailed definitions of the concepts of dynamic capabilities (innovation capability, absorptive capacity, and adaptability). Additionally, in section 3.2, "Variable Definition and Measurement", we elaborated on the measurement methods for the core variables and the control variables in detail. This explanation ensures that all variables can be derived. We have summarized the measurement methods for each variable in Table 2, "Definition of the main variables", presenting them in a clearer way.

**3.Comment:** Please provide a list of the keywords used for the construction of variables for example, digital transformation.

**3. Reply:** Thank you very much for providing such valuable feedback. We admit that our previous work overlooked the mapping of characteristic words for digital transformation. Therefore, we have referred to the research of Wu Fei et al. (2021) and drawn a structured characteristic word map for enterprise digital transformation, as shown in Fig 2. The structured feature-word map of firm digital transformation of the manuscript. This map can clearly illustrate which characteristic words are used to measure the digital transformation of enterprises, thus facilitating the calculation of the degree of digital transformation of enterprises through text analysis.

**4.Comment:** We have noted that the contribution of this manuscript in light of some other related research works such as 10.56028/aemr.11.1.512.2024; 10.1108/imds-09-2024-0907; 10.2991/978-94-6463-570-6_141; 10.3390/su17062652 has not been adequately. Therefore, kindly revise your manuscript to clearly indicate the contribution of this manuscript in light of the related published works identified above.

**4.Reply:** Thank you for raising such an important question. We attach great importance to it. First, we found all the four references and compared them one by one. We found that their research fields are roughly the same as ours, so there might be some repetitive parts in the manuscript. We have carried out de-duplication processing to avoid any controversial parts and strictly abide by academic ethics. In addition, for the parts that are of reference value to our research, we have made corresponding citations in the text. We guarantee that there is absolutely no plagiarism and all the materials are original. We also hope that this paper can make certain contributions to the research in related fields.

In addition, in accordance with the requirements of the PLOS ONE journal, we have revised the format of this manuscript and provided an amended funding statement. We have also made available all the original data necessary for replicating the research results and have cited all the figures and tables in the manuscript.

**Responses to reviewers (original comments by reviewers are in blue color)**

**Reviewer # 1:**

We sincerely appreciate your review of our manuscript and express our most heartfelt gratitude for your extremely detailed, professional and constructive comments! You not only precisely pointed out the deficiencies in the key parts of our research such as the introduction, theoretical analysis and hypotheses, research design and conclusion, but also provided specific, clear and highly instructive revision suggestions. These comments can help us more clearly recognize the limitations of the current version and the directions that need to be deepened. We fully agree with all your valuable opinions and have carefully and comprehensively revised them, providing detailed responses to each suggestion. The revised parts are highlighted in yellow in the manuscript. The following is a detailed response to each of your comments.

**1.Comment:** Introduction

(1)The definition of the "new quality productive forces " in the first paragraph is rather general. It is suggested to supplement its definition.

**(1)Reply:** Thank you very much for your detailed comments. Regarding the issue that the definition of "New quality productive forces (Nqpf)" is rather general, we referred to relevant studies and provided a detailed supplement to its definition in the introduction. Here, the core essence of Nqpf and the characteristics of its three elements are clearly defined.

(2)It only mentions that dynamic capabilities are a "promising mediators", but does not explain how it specifically act on the relationship between digital transformation and new quality productive forces. It is suggested to supplement this part, explain the reasons for choosing dynamic capabilities, and highlight the importance of dynamic capabilities.

**(2)Reply:** Thank you for your valuable suggestion, which has greatly contributed to the rigor of this paper. When we mentioned that dynamic capabilities are a “promising mediators”, we further elaborated that digital transformation influences dynamic capabilities, including innovation capability, absorptive capability, and adaptive capability. We also explained how dynamic capabilities affect the development of Nqpf. This clarifies how dynamic capabilities specifically function in the relationship between digital transformation and Nqpf, highlighting their significance. This is also a crucial reason why we chose dynamic capabilities as the mediating variable in this paper.

(3) Although the introduction mentions the impact of digital transformation on new quality productive forces, it does not provide a detailed explanation of the current situation, and specific effects of this transformation on the enterprise. This raises questions about the urgency and importance of the research, and makes it uncertain why it is important or what specific gaps it aims to fill in the current knowledge system. Therefore, I suggest specifying the important role of digital transformation and new quality productive forces , and explaining the research gap that the paper aims to fill.

**(3)Reply:** Thank you for your constructive suggestions. In the introduction, we proposed the significance of developing Nqpf to break through the prominent problems of "low efficiency, high consumption and high emissions" that enterprises still face in the process of transforming from scale expansion to quality and efficiency in the manufacturing industry. However, we found that the overall level of Nqpf in the manufacturing industry is still relatively low, with considerable room for development. Therefore, we put forward that digital transformation is an important factor in promoting the development of new quality productivityNqpf . This explains the urgency and importance of this research. It clarifies that how digital transformation affects Nqpf is not only of great significance for the high-quality development of manufacturing enterprises, but also for the development of advanced manufacturing in various countries. Based on the previous research of scholars, we summarized their limitations. Taking the manufacturing industry as the research object, we aim to fill the gap in empirical literature on the promotion of Nqpf development by digital transformation.

**2.Comment:** Theoretical analysis and research hypotheses

(1)This part has relatively little theoretical support. It is suggested to cite relevant theories to enrich this content and thereby enhance the depth of the paper.

**(1)Reply:** Thank you for offering such an important opinion, which has enhanced the rigor of this article. In Section 2.1, "Digital transformation of manufacturing and development of Nqpf", we started from the Resource-Based View to demonstrate that digital transformation is an important foundation for the development of Nqpf. Meanwhile, in Section 2.2, "The mediating role of dynamic capabilities", we cited the dynamic capability theory to prove that dynamic capabilities are an important channel through which digital transformation influences the development of Nqpf. The application of these two theories has further deepened the depth of the paper.

(2)The exposition of the Dynamic capabilities is relatively basic, only mentioning the definition of the Dynamic capabilities, without fully demonstrating the development and application of the theory in subsequent research. Therefore, I suggest citing more relevant research results to enrich this part of the content, supplementing more recent studies that have expanded or validated the theory, in order to reflect the forefront and completeness of the theory, and thus enhance the depth of the theory.

**(2)Reply:** Thank you for offering such valuable suggestions. In the revised manuscript, we have incorporated more relevant research findings to enrich the development and application of the dynamic capability theory in subsequent studies. This not only demonstrates new research on the role of dynamic capabilities in promoting enterprise performance but also indicates the new expansion of the dynamic capability theory in the field of artificial intelligence. Thus, it reflects the cutting edge and completeness of the dynamic capability theory and further enhances its depth.

(3)The dynamic capabilities directly adopt the three-dimensional division (innovation, absorption, and adaptation capabilities) of Wang & Ahmed (2007), but it is not explained why this framework was chosen instead of other classic classifications ,such as the three-dimensional division (sense,seize,transform) of Teece (2007).

**(3)Reply:** Thank you for offering such valuable suggestions, which have been of great help in improving this article. In the revised manuscript, we have focused on explaining why we chose the three-dimensional classification of dynamic capabilities proposed by Wang & Ahmed (2007). Considering that digital transformation is a process of adapting to the drastic changes in the external environment such as the impact of digital technology and the upgrading of market demands, and that Nqpf are centered on innovation driven, the three dimensional classification of dynamic capabilities proposed by Wang & Ahmed (2007) is more in line with our research content.

(4)The specific connotations of "innovation capacity", "absorptive capacity" and "adaptive capacity" have not been clarified. It is suggested to supplement their definitions

**(4)Reply:** Thank you for offering such an important suggestion. In the revised manuscript, we have further clarified the specific connotations of innovation capability, absorption capability and adaptive capability, thereby enriching the research content of this paper.

**3. Comment:** Research Design

(1)Only "database" was mentioned without specifying the exact name of the database. It is recommended to clarify the source of the data.

**(1)Reply:** Thank you very much for pointing out the omissions in this article. This was a serious mistake on our part. In the revised manuscript, we have not only specified the exact name of the database but also clearly indicated the source of data for each variable as required by the journal.

**4. Comment:** Conclusion

(1)The literature support for the theoretical exposition in this part is slightly insufficient. It is suggested to supplement relevant literature to enhance the rigor and scientific nature of the argument.

**(1)Reply:** Thank you for your constructive suggestions. In each conclusion discussion, we have added relevant literature to enhance the rigor and scientific nature of the arguments. A total of 16 related references have been added.

(2)The conclusion section only briefly lists a few relationship results obtained from the research, which seems relatively thin. Therefore, I suggest briefly explaining the findings of this study and explaining the differences and connections between the results of this study and previous research. Combining the analysis results with existing research, further summarizing, improving, and enhancing the conclusion content.

**(2)Reply:** Thank you for raising such an important point. This is indeed a weak point of this article. Through this revision, when presenting the empirical result that the digital transformation of the manufacturing industry has significantly enhanced the level of Nqpf, we first explained the research findings, then discussed the differences and connections with previous studies in terms of research objects and the selection of Nqpf indicators, and finally comprehensively discussed and clarified the research significance of this article, highlighting its research contributions. Secondly, when presenting the research result that the innovation ability, absorption ability and adaptability of enterprises play a partial mediating role in the process of promoting the development of Nqpf through the digital transformation of the manufacturing industry, we immediately provided an explanation for this result, then discussed the differences between the mediating mechanisms in previous studies on digital transformation and Nqpfand this article, and once again verified a research conclusion from previous studies. Finally, we comprehensively discussed and clarified the important research significance of taking dynamic capabilities as the mediating mechanism. In addition, when presenting the result that the digital transformation has a greater enhancing effect on the level of Nqpf in non-state-owned and high-tech manufacturing enterprises, we first provided an explanation for this result. Then, by comparing the previous studies and the newly added life cycle heterogeneity test in this article, we discussed the differences and connections with previous research. Finally, we comprehensively discussed and clarified the important research significance of the heterogeneity test.

(3)The “Practical significance” section states that “fostering a digital mindset and innovation capabilities”, this is a common practice. Therefore, I suggest proposing specific operational plans.

**(3)Reply:** Thank you for your valuable research, which is of great significance to the improvement of the quality of this paper. In Section 6.3, “Practical Significance”, we have provided more specific and feasible suggestions for the development of manufacturing enterprises based on the research conclusions. We have refined the previous broad and vague suggestions, making them more detailed and concrete. For instance, we recommend the adoption of digital technologies such as AI and big data to analyze and optimize production plans and inventory control, and the establishment of an "Innovation Fund" to encourage employees to propose practical innovative ideas around cost reduction and efficiency improvement or new product development. Such suggestions can better assist enterprises in implementing digital transformation, thereby promoting the development of Nqpf.

**Reviewer # 2:**

Thank you very much for taking the time to review our manuscript and provide professional and practical suggestions! Your views on the rigor of the research methods, the depth of data analysis, and the integration of theory and practice have been of great help to us. Your advice has pointed out the key directions for improving the logicality of our research, the reliability of the results, the depth of analysis, and the practical value. We fully accept all your suggestions and have made corresponding revisions in the revised manuscript, striving to enhance the quality of the article. The revised parts are highlighted in yellow in the manuscript. Once again, we sincerely thank you for spending your precious time on a thorough review and providing such insightful and specific guidance! The following is a detailed response to each of your comments.

**1.Comment:** Refine Hypothesis Development: Beyond existing hypotheses, elaborate on the nuanced dimensions of how digital transformation affects Nqpf, specifically detailing the mediating roles of innovation, absorptive, and adaptive capacities. The current hypothesis development is overly verbose and lacks logical coherence. Each hypothesis must be grounded in explicit theoretical foundations (e.g., innovation theory, resource-based view) with clear reasoning.

**1.Reply:** Thank you very much for your important suggestion, which has enhanced the rigor of this article. In Section 2.1, "Digital transformation of manufacturing and development of Nqpf", based on the Resource-Based View, we elaborated in detail on the impact of digital transformation on New quality productive forces (Nqpf). Meanwhile, we also discussed in detail the influence of digital transformation on the micro dimensions of Nqpf, including new quality labourers, new quality means of labour, new quality objects of labour, and ecological environment, thereby proposing the hypothesis that digital transformation can promote Nqpf. In Section 2.2, "The mediating role of dynamic capabilities", based on the dynamic capability theory, we elaborated on how digital transformation affects the innovation capability, absorptive capability, and adaptive capability of enterprises, thereby promoting the development of Nqpf. Through strict reasoning and argumentation, the logical coherence of this article has been improved.

**2.Comment:** Strengthen Mediation Analysis & Methodology: While the model references Jiang T's two-step mediation test, the hypotheses inadequately elaborate on how the mediating variables (innovation/absorptive/adaptive capacities) influence Nqpf. Given the limited literature explicitly linking these three specific capacities to Nqpf, it is recommended to adopt a three-step mediation test to mitigate severe endogeneity concerns like spurious regression. Key references include:

[1] Chin, T., Li, Z., Huang, L., & Li, X. (2025). How artificial intelligence promotes new quality productive forces of firms: a dynamic capability view. Technological Forecasting & Social Change, 216.

[2] Tian, H., Zhao, L., Li, Y., Wang, W. (2023). Can enterprise green technology innovation performance achieve “corner overtaking” by using artificial intelligence? —Evidence from Chinese manufacturing enterprises. Technological Forecasting & Social Change, 194, 122732.

**2.Reply:** Thank you for offering such valuable suggestions and pointing out the areas where our paper lacks rigor. When we were conducting the theoretical analysis, we also found that there is a scarcity of literature that links the three specific dynamic capabilities with Nqpf. This is in conflict with Jiang T's two-step mediation method, which requires a large amount of literature to explain how the three specific dynamic capabilities promote the development of Nqpf. Therefore, we are once again grateful for your valuable suggestions and the corresponding references you provided. Based on the research of Tian et al., we reconstructed the three-step mediation mechanism model and reexamined the mediating role of dynamic capabilities. The research results are consistent with the previous conclusion, which also indicates the reliability of our research conclusion. In addition, we used the Bootstrap test method to test the mediation path, further ensuring the robustness of the intermediate mechanism.

**3.Comment:** Enhance Robustness Checks for Endogeneity: The endogeneity treatment section should be strengthened beyond basic tests. Conduct additional robustness checks, such as shortening the sample period or employing alternative regression models, to convincingly demonstrate model stability and reliability.

**3.Reply:** Thank you very much for your constructive suggestions. Based on the original robustness test, we added a Tobit model to reexamine the impact of digital transformation in manufacturing on Nqpf. We found that after changing the regression model, digital transformation in manufacturing has a significant positive impact on the level of Nqpf. This once again proves the stability and reliability of the model.

**4.Comment:** Deepen Heterogeneity Analysis: The current analysis is superficial. Provide meaningful insights by rigorously comparing the effects across diverse firm types (e.g., technology-intensive vs. labor-intensive firms, different lifecycle stages). Identify key differences and commonalities in how digital transformation impacts Nqpf within these groups, offering concrete references for corporate digital policy and practice. For methodology and framing, see:

[1] Chin, T., Li, Z., Huang, L., & Li, X. (2025). How artificial intelligence promotes new quality productive forces of firms: a dynamic capability view. Technological Forecasting & Social Change, 216.

**4.Reply:** Thank you for offering such valuable suggestions, which are of great significance to the improvement of the quality of this manuscript. After reading the literature you provided, we found that the quality of this paper is very high and the research is rigorous and standardized. We have also cited this paper in our manuscript. In addition, based on this reference, we have decided to delete the previous test of regional heterogeneity of enterprises and add a test of enterprise life cycle heterogeneity. The purpose is to deepen the heterogeneity analysis and provide more meaningful references for the digitalization policies and practices of enterprises. Through the analysis of enterprise life cycle heterogeneity, we found that in manufacturing enterprises in the growth stage, the promotion effect of digital transformation on Nqpf is more obvious, followed by mature enterprises, and finally enterprises in the decline stage.

**5.Comment:** Revise Conclusion Section & Enhance Implications: The heading "6. Conclusion" is inadequate; rename it "6. Discussion and Conclusion". Significantly strengthen this section by deriving specific, actionable recommendations for both enterprises (on implementing digital transformation strategies to boost Nqpf) and government policymakers, grounded in the theoretical and empirical findings.

**5.Reply:** Thank you for your detailed suggestions. We have revised the original title 6 to "6. Discussion and Conclusion". Additionally, we have rediscussed the research conclusions of this article and provided a thorough discussion. Finally, in section 6.3 on practical significance, based on the research conclusions, we have offered more specific and feasible suggestions for the development of manufacturing enterprises. We have refined the previous broad and vague suggestions, making them more detailed and concrete. For instance, we recommend the adoption of digital technologies such as AI and big data to analyze and optimize production plans and inventory control, and the establishment of an "innovation fund" to encourage employees to propose practical innovative ideas centered around cost reduction and efficiency improvement or new product development. Such suggestions can better assist enterprises in implementing digital transformation, thereby promoting the development of Nqpf.

**6.Comment:** Improve Overall Structure, Language, and Formatting:Optimize the logical flow and transitions between sections for clarity and reader comprehension.Ensure consistent formatting in all tables and figures. For example, maintain three decimal places for Adjusted R² values unif-ormly (Table 6, Column 2 currently shows four) to enhance professionalism.Thoroughly polish the English language throughout the manuscript.

**6.Reply:** Based on your suggestions, we have reorganized the logic of the paper, added some new transitional words to make the transitions between paragraphs smoother. Besides, we have checked all the formats in the paper to ensure consistency. Finally, we have polished the entire manuscript to improve its overall fluency.
