# Supplementary material for: Digital transformation, dynamic capabilities and new quality productive forces: Empirical data from listed Chinese manufacturing companies
Source: PLoS One. 2026 Mar 24;21(3):e0342917. doi: 10.1371/journal.pone.0342917 (PMC13012489; doi:10.1371/journal.pone.0342917)
Supplement: Attachment — Submitted filename: Response_to_Reviewers_auresp_2.docx [file pone.0342917.s003.docx]

**Reply to the Second External Review Opinion**

**Responses to editor (original comments by editor are in blue color)**

We sincerely appreciate the valuable comments you provided during the review process. These comments are of great significance in improving the quality of our manuscript. We are particularly grateful for your meticulous attention to the accuracy of the references and the details of the paper content. These comments not only helped us identify the areas that need improvement, but also deepened our understanding of the rigor and standardization of academic research. We have made revisions based on each of your comments and have accordingly improved the manuscript. The revised parts are highlighted in yellow in the manuscript. Below are detailed responses to each of your comments.

**Journal Requirements**

**1.Comment:** If the reviewer comments include a recommendation to cite specific previously published works, please review and evaluate these publications to determine whether they are relevant and should be cited. There is no requirement to cite these works unless the editor has indicated otherwise.

**1.Reply:** Thank you for your careful reminder. In the first external review opinion, Reviewer 2 mentioned two important references. We carefully read these two papers and indeed found that they are highly relevant to this thesis. When constructing the research model, we referred to the paper by Tian et al. (2023). However, when conducting the test of heterogeneity in the enterprise life cycle, we did not refer to the paper by Chin et al. (2025) provided by Reviewer 2 because its method of dividing the enterprise life cycle was not suitable for this study. Therefore, this thesis referred to the more appropriate research by Li et al. (2011). In the second external review opinion, no expert raised such an opinion.

39.Tian H, Zhao L, Yunfang L, et al. Can enterprise green technology innovation performance achieve “corner overtaking” by using artificial intelligence?—Evidence from Chinese manufacturing enterprises. Technological Forecasting and Social Change. 2023;194:122732. <https://doi.org/10.1016/j.techfore.2023.122732>

44.Li YH, Li Z, Tang SL. Corporate Life-cycle, Corporate Governance and Corporate Capital Allocation Efficiency. Nankai Business Review. 2011;14(03):110-121. https://doi.org/10.3969/j.issn.1008-3448.2011.03.013

**2.Comment:** Please review your reference list to ensure that it is complete and correct. If you have cited papers that have been retracted, please include the rationale for doing so in the manuscript text, or remove these references and replace them with relevant current references. Any changes to the reference list should be mentioned in the rebuttal letter that accompanies your revised manuscript. If you need to cite a retracted article, indicate the article’s retracted status in the References list and also include a citation and full reference for the retraction notice.

**2.Reply:** Thank you very much for your valuable suggestions. According to the journal's requirements, we carefully reviewed the reference list and found no cases of citations of papers that have been withdrawn. We ensured its completeness and accuracy. At the same time, when modifying 6.2 Theoretical significance, we re-quoted 5 new references and highlighted them in yellow at the end of the reference list.

**Additional Editor Comments**

**1.Comment:** Introduction – Clarify the connection between general digital technologies (e.g., AI, big data, cloud computing) and the manufacturing field; add specific examples such as Industrial IoT or smart factories.

**1. Reply:** Thank you for your very constructive suggestion. In the first paragraph of the introduction, we discussed the deep penetration and wide application of advanced technologies such as artificial intelligence, big data, and cloud computing. To enhance the discussion, we included two real examples from manufacturing enterprises. One is that China's Xiaomi Automobile in the automotive manufacturing sector achieved efficient and automated production through an intelligent factory. The other is that the Electronic Works Amberg (EWA) in the electronic manufacturing sector utilized the MindSphere industrial Internet of Things operating system to achieve full-process data collection and intelligent control. Additionally, when exploring the strategic and future industries brought about by digital technologies, we listed the rapid development of embodied intelligent robots and quantum information industries, which are currently popular. Through these examples, we further proved that the digital transformation of manufacturing is rapidly promoting the development of new quality productive forces (Nqpf) of enterprises.

**2.Comment:** Empirical Analysis – Ensure that all tables are correctly formatted and presented; for instance, Table 5 appears in Chinese in the text.

**2.Reply:** Thank you for raising such detailed questions. We are extremely sorry that due to our carelessness, such a trivial mistake occurred in the text. We have revised the issue in Table 5 and carefully checked all the formatting of the entire text to ensure that such a trivial error will not occur again.

**3.Comment:** Discussion and Conclusion – Make the theoretical contributions more specific, clearly identify gaps in existing literature, and explain how your study fills them.

**3.Reply:** Thank you for your very important suggestion. We also noticed that the previous part about theoretical significance seemed rather vague, failing to highlight the unique value of the research. Firstly, this article explores and enriches the existing research on the outcome effects of digital transformation, and deepens the first theoretical significance of the industry adaptability of digital transformation promoting the development of Nqpf. It explains that the empirical evidence of existing research on how enterprises at the micro level cultivate Nqpf through digital transformation is still relatively scarce. At the same time, existing literature mostly uses samples of all industry listed companies and ignores the unique industry attributes of manufacturing enterprises, thereby highlighting the theoretical value of this study. Secondly, this article explores the significance of the theoretical framework of "digital transformation - dynamic capability - Nqpf", and explains that existing research mostly focuses on the mediating paths at the enterprise financial or governance level, lacking in-depth exploration of the enterprise capability transmission mechanism.Finally, this article explores the differentiated impacts of digital transformation on the Nqpf of enterprises with different characteristics, and explains that existing research still lacks theoretical exploration on which enterprises can better cultivate Nqpf through digital transformation. Therefore, in this revision, we have made the theoretical significance more specific and compared it with existing research, further highlighting the theoretical value of this study.

**Responses to reviewers (original comments by reviewers are in blue color)**

**Reviewer # 1:**

We sincerely thank you for your review of our paper, and we express our most heartfelt gratitude for your extremely detailed, professional and constructive comments! You not only accurately pointed out the deficiencies in the key parts of our research (such as the introduction, formatting, and theoretical significance), but also provided specific, clear and highly instructive revision suggestions. These comments can help us more clearly recognize the limitations of the current version and the directions that need to be further deepened. We fully agree with all your valuable opinions and have carefully and comprehensively made the revisions. We have given detailed responses to each suggestion. The revised parts are marked in yellow in the paper. Here are the detailed responses to each of your comments.

**1.Comment:** Introduction

The terms such as “artificial intelligence, big data and cloud computing” mentioned in the first paragraph are all very macroscopic and general expressions, lacking a close integration with the specific field of “manufacturing”. Adding one or two specific cases or directions of digitalization in the manufacturing field (such as industrial Internet of Things, smart factories) will make the discussion more solid.

**1.Reply:** Thank you for your very constructive suggestion. In the first paragraph of the introduction, we discussed the deep penetration and wide application of advanced technologies such as artificial intelligence, big data, and cloud computing. To enhance the discussion, we included two real examples from manufacturing enterprises. One is that China's Xiaomi Automobile in the automotive manufacturing sector achieved efficient and automated production through an intelligent factory. The other is that the Electronic Works Amberg (EWA) in the electronic manufacturing sector utilized the MindSphere industrial Internet of Things operating system to achieve full-process data collection and intelligent control. Additionally, when exploring the strategic and future industries brought about by digital technologies, we listed the rapid development of embodied intelligent robots and quantum information industries, which are currently popular. Through these examples, we further proved that the digital transformation of manufacturing is rapidly promoting the development of new quality productive forces (Nqpf) of enterprises.

**2.Comment:** Empirical analysis

It is not certain whether it is a format issue. The last column of Table 5. Robustness test results in the text is shown in Chinese. It is recommended to check the article.

**2.Reply:** Thank you for raising such detailed questions. We are extremely sorry that due to our carelessness, such a trivial mistake occurred in the text. We have revised the issue in Table 5 and carefully checked all the formatting of the entire text to ensure that such a trivial error will not occur again.

**3. Comment:** Discussion and conclusion

The description of the theoretical significance section Such as “enriching the research on the antecedent factors of Nqpf and extending the theoretical boundaries of existing digital transformation” seems rather vague and fails to highlight the unique value of the research. Suggestions should be more specific, such as what has been “deepened” and where has been “expanded”. It is necessary to clearly point out the specific gaps or deficiencies in the existing literature in explaining the issue of “how digital transformation affects Nqpf”. For instance, was the micro-mechanism overlooked, or was there a lack of attention paid to the specific field of manufacturing? Then explain how your research fills this gap.

**3.Reply:** Thank you for your very important suggestion. We also noticed that the previous part about theoretical significance seemed rather vague, failing to highlight the unique value of the research. Firstly, this article explores and enriches the existing research on the outcome effects of digital transformation, and deepens the first theoretical significance of the industry adaptability of digital transformation promoting the development of Nqpf. It explains that the empirical evidence of existing research on how enterprises at the micro level cultivate Nqpf through digital transformation is still relatively scarce. At the same time, existing literature mostly uses samples of all industry listed companies and ignores the unique industry attributes of manufacturing enterprises, thereby highlighting the theoretical value of this study. Secondly, this article explores the significance of the theoretical framework of "digital transformation - dynamic capability - Nqpf", and explains that existing research mostly focuses on the mediating paths at the enterprise financial or governance level, lacking in-depth exploration of the enterprise capability transmission mechanism.Finally, this article explores the differentiated impacts of digital transformation on the Nqpf of enterprises with different characteristics, and explains that existing research still lacks theoretical exploration on which enterprises can better cultivate Nqpf through digital transformation. Therefore, in this revision, we have made the theoretical significance more specific and compared it with existing research, further highlighting the theoretical value of this study.

**Reviewer # 3:**

Although you did not offer any comments on this study this time, we are extremely grateful for your previous insightful views on the rigor of the research methods, the depth of the data analysis, and the degree of integration of theory and practice. These have been of great help to us. Once again, we thank you for your recognition of this study. We have further reviewed our paper, including details such as formatting and language, to ensure it meets the requirements for publication. The revised parts are marked in yellow in the paper. Below are detailed responses to each of your comments.

**1.Comment:** This pape presents an insightful exploration into the impact of digital transformation on new quality productive forces (Nqpf) within the manufacturing industry. The research adopts a novel perspective and employs rigorous scientific methods. Through empirical analysis, the author effectively demonstrates the significant role of digital transformation in enhancing Nqpf, innovatively introducing dynamic capabilities as mediating variables to elucidate their underlying mechanisms. The paper is well-structured, logically coherent, and backed by reliable data sources, offering in-depth analysis. Notably, the theoretical framework, integrating Resource-Based View and Dynamic Capabilities Theory, provides solid theoretical support. Furthermore, the heterogeneity analysis enriches the research content, enhancing the generalizability of the conclusions. This study not only offers theoretical guidance for digital transformation in manufacturing but also provides valuable insights for policymakers, holding substantial academic and practical significance.

**1.Reply:** We sincerely thank the reviewers for their meticulous review and professional, constructive evaluations of this paper. We also sincerely appreciate your full affirmation of the research topic, analytical perspective, research methods, and academic value of this paper! This paper focuses on the research core of enabling Nqpf development through digital transformation in the manufacturing industry. We attempt to establish an analytical framework integrating the resource-based view and the dynamic capability theory, introduce dynamic capability as an intermediary variable to reveal the internal mechanism of the two, and expand the research boundaries through heterogeneity analysis to enhance the universality of the conclusions. Throughout the research process, we have always strived for the rigor of empirical analysis and the completeness of theoretical logic. Your recognition not only affirms the research ideas and results of this paper, but also points out the direction for our subsequent revision and improvement work. In the future, we will strictly follow the review opinions, further optimize and polish the details of the manuscript, continuously improve the argumentation logic, and solidify the research conclusions, striving to make the research content more profound and rigorous. Once again, we express our most sincere gratitude to the reviewers for your valuable time and professional academic guidance!
